# Employing lytic phage-mediated horizontal gene transfer in *Lactococcus lactis*

**DOI:** 10.1371/journal.pone.0238988

**Published:** 2020-09-14

**Authors:** Barbara Marcelli, Harma Karsens, Mark Nijland, Ruben Oudshoorn, Oscar P. Kuipers, Jan Kok

**Affiliations:** Department of Molecular Genetics, Groningen Biomolecular Sciences and Biotechnology Institute, University of Groningen, Groningen, The Netherlands; Technical Educational Institute of Peloponnese, GREECE

## Abstract

*Lactococcus lactis* is a lactic acid bacterium widely used as a starter culture in the manufacture of dairy products, especially a wide variety of cheeses. Improved industrial strains would help to manufacture better food products that can meet the industry’s and consumer’s demands with respect to e.g. quality, taste, texture and shelf life. Bacteriophage infection of *L*. *lactis* starter cultures represents one of the main causes of fermentation failure and consequent economic losses for the dairy industry. In this study, however, we aim at employing bacteriophages for beneficial purposes. We developed an experimental setup to assess whether phage-mediated horizontal gene transfer could be used to enhance the genetic characteristics of *L*. *lactis* strains in accordance with the European law regarding the use of genetically modified organisms (GMOs) in the food industry.

Although we could not show the transfer of chromosomal DNA we did successfully transduce two dissimilar plasmids from *L*. *lactis* strain MG1363 to one of its derivatives employing three different lactococcal bacteriophages.

## Introduction

*Lactococcus lactis* is the most-used lactic acid bacterium (LAB) in the dairy industry, where it is employed as starter culture for the manufacture of products such as buttermilk, quark, and especially a wide variety of hard and soft cheeses [1]. It is mainly responsible for the rapid acidification of the raw milk, and for the improvement of the shelf life and the development of organoleptic qualities of the fermented foods [2–4]. Many companies nowadays use predefined combinations of different *L*. *lactis* strains as starters for the production of specific types of dairy products in order to obtain distinct end products that meet consumers’ wishes and preferences [5]. Consequently, a great effort is constantly put into the development of new and enhanced industrial starter strains with specific metabolic characteristics, with the aim of steering the fermentation process towards the desired final product. Recombinant DNA technology, which is routinely used in research laboratories, would represent a perfect strategy for selective genotypic and phenotypic improvement of *L*. *lactis* because of its unprecedented accuracy and reliability. However, strains obtained by using this approach are considered as genetically modified organisms (GMOs). As the current European Union legislation forbids directly employing GMOs in the food industry, it is important to develop alternative methods to change or enhance the genomes of industrial LAB strains at will [6, 7]. Techniques such as random mutagenesis, adaptive mutation and dominant selection, are nowadays in some instances successfully adopted in dairy industrial settings [8]. All the aforementioned strategies, however, display certain drawbacks such as the risk of introducing undesirable mutations in the strains of interest, the inability to acquire substantial amounts of new genetic material, or the relatively lengthy process by which they allow isolating LAB strains with enhanced metabolic characteristics [8]. For this reason, there is still an urgent need to establish new and reliable tools that can be used to modify the genomic content of LAB, including *L*. *lactis*, for industrial purposes. The latter issues have drawn renewed attention to non-GMO methodologies like natural Horizontal Gene Transfer (HGT). HGT is defined as the movement of genetic material between two bacterial cells in a way different than vertical gene transfer (from parent to offspring). Naturally occurring HGT mechanisms include (i) conjugation, (ii) transformation, (iii) bacteriophage transduction, (iv) and mobile genetic elements (MGEs) action [9, 10]. Recently also the formation of nanotubes has been proposed as HGT mechanism [11].

Bacteriophages are viruses that infect bacterial cells. They are found in all types of environments and they contribute to HGT via transduction [12]. Phages of *L*. *lactis* have received a lot of attention in the past three decades due to their abundant and destructive presence in the dairy setting [13]. All known bacteriophages infecting *L*. *lactis* belong to the *Caudovirales* order as they possess a double stranded DNA (dsDNA) genome that is packaged into a proteinaceous capsid [14].

They can undergo two main replication modes, namely the lytic or lysogenic cycle [15]. In the first case, the virus actively replicates inside the host cytoplasm and when the new viral progeny is assembled, the host cell bursts releasing the progeny into the environment. Lysogenic replication is typical of temperate phages. The genome of these phages can, after infection integrate into the chromosome of the infected cell taking the name of prophage. The cell, thus transformed into a so-called lysogen, replicates the inserted DNA together with the rest of its genome during normal cell growth and division, passing down the prophage to its progeny [15]. External agents imposing a stress on the lysogen (e.g. antibiotic treatment or UV radiation) can induce excision of the prophage, which will then start the lytic replication cycle and escape from its compromised host [15]. In both instances, during active replication, the newly synthesized genomes, need to be packaged into empty capsids to proceed with virion assembly. Two distinct methods of DNA packaging, *cos* and *pac*, have been recognized in dsDNA bacteriophages [16]. In *cos*-type phages, unit lengths of bacteriophage genome in the replication-derived concatemers are separated by a specific nucleotide sequence called *cos* (cohesive end site). A phage-encoded enzyme called terminase recognizes and binds to the first of these *cos* sequences and packages the viral DNA inside an empty capsid via an ATP-dependent mechanism until it encounters the next *cos* sequence. At this point, it cuts the concatemer triggering the closure of the capsid filled with a unit-length of the phage genome. In *pac*-type phages, packaging follows the so-called headful mechanism. In this case, packaging starts with the terminase recognizing and binding to a nucleotide sequence called *pac* on the concatemer of phage genomes. Packaging stops when the capsid is filled to its maximum physical capacity, causing each phage particle to contain 102% to 110% of the viral genome [16].

The above described processes of phage DNA replication, integration/excision and packaging allow for errors to be made, and consequently for genetic flexibility. Indeed, bacteriophages have been shown to contribute to HGT through a process called transduction, which starts with fragments of host DNA being erroneously packaged instead of, or together with the phage DNA [15]. The resulting bacteriophage is called a transducing particle, as it will more often than not be defective as a phage. The transducing particle has an intact tail and can adsorb and inject its DNA content into a sensitive host cell which is, upon successful establishment of the injected DNA (e.g. by integration into the recipient genome and expression), genetically altered (transduced) [15].

In generalized transduction, any part of the host chromosomal or plasmid DNA can be packed inside a phage capsid and transferred to a second host cell. In this case the transducing particle only contains bacterial DNA [17]. Specialized transduction is typically performed by temperate phages. When a prophage is induced and excises from the host chromosome, a fragment of neighbouring chromosomal DNA can be accidentally excised as well, packed into a nascent phage particle and transferred to a new host [15]. Several cases of phage-mediated HGT have been reported in *L*. *lactis* and other LAB [18–22]. More recently, Ammann and co-workers have shown that plasmid DNA can be transduced from *Streptococcus thermophilus* to *L*. *lactis* [23]. This finding demonstrates that phage transduction can, in certain occasions, cross species borders and sets new perspectives on the possible application of this phenomenon in laboratory and industrial settings.

Based on this knowledge, we decided to investigate the feasibility of using bacteriophage transduction in *L*. *lactis* as a tool for genome editing. Our goal was to provide a proof of concept that bacteriophage transduction may be used in an industrial setup to mobilize plasmid and chromosomal bacterial DNA among *L*. *lactis* strains with the aim of improving their metabolic activities. As the already reported examples of successful HGT mediated by bacteriophage transduction in *L*. *lactis* involved the use of lysogenic bacteriophages, we tested the transduction ability of strictly lytic lactococcal phages and show some successful cases.

## Materials and methods

### Bacterial strains, bacteriophages and culture conditions

The bacterial strains and plasmids used in this study are presented in Table 1, bacteriophages are listed in Table 2. All *L*. *lactis* strains were grown at 30°C in M17 medium (BD—Becton, Dickinson and Company, Franklin Lakes, NJ, USA) supplemented with 0.5% glucose (GM17) or 0.5% lactose (LM17). Erythromycin, streptomycin, rifampicin and chloramphenicol were used, when needed, at final concentrations of 5 μg/ml, 200 μg/ml, 50 μg/ml, and 5 μg/ml, respectively. Colony forming units (CFU) were estimated by plating serial dilutions of a liquid culture of the strain to be tested on GM17 agar (1.5%) plates.

**Table 1:** List of bacterial strains and plasmids used in this work.

*Lactococcus lactis* strain or plasmid	Description*	Reference
**MG1363**	*L*. *lactis* subsp. *cremoris*; plasmid-free derivative of NCDO712	[39]
**MG1614**	*L*. *lactis* subsp. *cremoris*. Str^r^ and Rif^r^ derivative of MG1363	[39]
**MG1363 transposon library**	Random transposon insertion library; Em^r^ and GFP^+^	Laboratory collection
**pVI110**	Em^r^, pBR322*ori*, P_junc_	[29]
**pVI129**	Ap^r^ Cm^r^, pBR322*ori*, pIP501*ori*, cop^+^, containing P_hlbA_-IS*1223*ΔIR	[29]
**pVE6007**	Cmr, Ts derivative of pWV01	[31]
**pSEUDO::Pusp45-sfgfp(Bs)**	Em^r^, pSEUDO derivative, containing *Pusp45-sfgfp(Bs)*	[30]
**pLP712**	Lac^+^, Prt^+^	[32]
**pNZ8048**	*L*. *lactis* nisin-controlled gene expression vector; Cm^r^	[40]
**pGKV552**	*L*. *lactis* vector containing the entire *prtP* gene and the *prtM* gene lacking three 3’-codons; Em^r^	[41]

* = Str^r^: Streptomycin resistance, Rif^r^: Rifampicin resistance, Em^r^: Erythromycin resistance; Ap^r^: Ampicillin resistance, Cm^r^: chloramphenicol resistance, GFP: Green Fluorescence Protein, *cop*^+^: containing the copy number controlling *cop*R gene, Lac^+^: containing the intact lactose utilization operon, Prt^+^: containing the proteinase and maturation genes (*prtP*, *prtM*) and peptidase F (*pepF*) genes.

**Table 2:** List of bacteriophages used in this study.

*L*. *lactis* bacteriophage	Phage species	Origin	Year of isolation	Reference	GenBank Accession number	Infection of *L*. *lactis* MG1363^a^
**CHPC52**	936	Unknown	1997	Internal laboratory data	MN689519	
**CHPC116**	c2	USA	1989	Internal laboratory data	MN689507	
**CHPC122**	c2	UK	1990	Internal laboratory data	MN689512	
**CHPC129**	936	UK	1990	Internal laboratory data	MN689514	
**CHPC134**	c2	UK	1990	Internal laboratory data	MN689515	✓
**CHPC148**	Bk5t	UK	1990	Internal laboratory data	MN689516	
**CHPC361**	936	Unknown	1988	Internal laboratory data	MN689517	
**CHPC362**	936	Unknown	1988	Internal laboratory data	MN689518	
**CHPC781**	936	Denmark	1997	Internal laboratory data	MN689520	
**CHPC958**	936	Australia	1997	Internal laboratory data	MN689522	
**CHPC972**	c2	USA	2002	Internal laboratory data	MN689528	
**CHPC973**	c2	USA	2002	Internal laboratory data	MN689529	
**CHPC974**	Bk5t	USA	2002	Internal laboratory data	MN689530	
**CHPC1020**	c2	Australia	2004	Internal laboratory data	MN689505	
**CHPC1161**	c2	USA	2009	Internal laboratory data	MN689506	
**CHPC1170**	c2	USA	2009	Internal laboratory data	MN689508	✓
**CHPC1182**	c2	USA	2010	Internal laboratory data	MN689510	
**CHPC1183**	c2	USA	2010	Internal laboratory data	MN689511	✓
**CHPC967**	c2	USA	2002	Internal laboratory data	MN68952	
**CHPC1175**	Bk5t	USA	2009	Internal laboratory data	MN689509	
**CHPC1242**	c2	Germany	2013	Internal laboratory data	MN689513	
**CHPC836**	Bk5t	France	1998	Internal laboratory data	MN689521	
**CHPC959**	936	USA	2002	Internal laboratory data	MN689523	
**CHPC964**	936	USA	2002	Internal laboratory data	MN689524	
**CHPC965**	936	USA	2002	Internal laboratory data	MN689525	
**CHPC971**	1706	USA	2002	[47]	MK779875	
**CHPC966**	c2	USA	2002	This work	MN689526	✓
**5171F**	c2	The Netherlandds	2006	This work	MN689503	✓
**5105F**	c2	Germany	2007	This work	MN689504	✓

^a)^ ✓, the phage can propagate on MG1363; no entry, the phage cannot propagate on MG1363.

Skim milk media, supplemented with 0.5% glucose, was prepared for testing proteinase PrtP activity. The media was prepared by mixing a 10% skim milk solution and a 2.4% agar/1.5% sodium citrate solution at pH9, in a 1:1 (v/v) ratio. The skim milk solution was separately sterilized by autoclaving at 120°C for 5 min.

Bacteriophages were initially propagated on their sensitive industrial strains by infecting, with a single plaque, a 10 ml culture in its early exponential growth phase (Optical density at 600 nm; OD_600_ 0.3–0.5) in LM17 containing 10 mM CaCl_2_ and 10 mM MgCl_2_. Samples were incubated at 30°C until visible culture lysis occurred, filter-sterilized using a 0.45 μm filter (MinistartNML, Sartorius, Germany) to eliminate cells and cell debris, and stored at 4°C until further use. Bacteriophage titres were determined by counting plaque forming units (PFU) using the double-layer plaque assay as previously described [24] with the following modifications: bottom and top agar layers contained 1% and 0,4% agar, respectively. CaCl_2_ was added to the media at the final concentration of 10 mM. Glycine was added at the final concentration of 0.5% (wt/vol) to facilitate plaque visualization as previously reported [25]. Bacteriophage lysates were diluted in TBT buffer (100 mM NaCl, 50 mM Tris-HCl, 10 mM MgCl_2_, [pH7]). 200 μl of an overnight sample of the bacterial strain and 10 μl of properly diluted phage lysate was mixed with 4 ml of top agar, the mix was poured on a solidified bottom agar plate. The plates were incubated overnight at 30°C and subsequently examined for the presence of phage-derived plaques.

### Bacteriophage species analysis and host range assay

The host range of the bacteriophages was assessed via a spot test using the double-layer plaque assay as previously described [24]. A phage lysate was serially diluted with 10-fold increments in TBT buffer and 10 μl of either undiluted, 10-fold diluted and 100-fold diluted phage samples were spotted onto the solidified overlay only containing 200 μl of the bacterial strain to test, and allowed to dry for 30 min at RT. The plates were incubated overnight at 30°C and then examined for the presence of spots of bacterial lysis and the appearance of phage-derived plaques in the spot areas. If lysis occurred at the position of the 100-fold diluted sample, the bacterial strain under study was considered to be possibly sensitive to the phage. In that case, the phage and bacterial strain were subjected to the plaque assay described above in order to check for the appearance of phage-derived plaques and to confirm the ability of the phage to propagate on the bacterial strain. All experiments were conducted in triplicate.

Bacteriophage species was assigned using two multiplex PCR methods as previously reported [26, 27].

### DNA techniques

Plasmids pNZ8048 or pGKV552 were introduced by electrotransformation into *L*. *lactis* MG1363 [28] using a Gene Pulser electroporation system (Bio-Rad, Richmond, CA, USA). Selection of transformants was performed on GM17 plates containing 0.5 M sucrose (SGM17) and 5 μg/ml of Cm or 5 μg/ml Ery, respectively, for the two plasmids. Plasmid pNZ8048 isolated from *L*. *lactis* MG1363 (pNZ8048) and from the transductants was digested using the restriction enzymes SalI and HindIII, while plasmid pGKV552 from donor and transductants was digested using the restriction enzymes XbaI and BglII (all fast-digest enzymes were from Thermo Fisher Scientific, Walthman, MA, USA).

### Experimental setup of phage transduction

A bacteriophage was first propagated on the donor strain carrying an antibiotic resistance gene on a plasmid or integrated in the chromosome to obtain a lysate as follows: 10 ml of culture of the donor strain in the early exponential growth phase in GM17 containing 10 mM CaCl_2_ and the appropriate antibiotic was infected at a multiplicity of infection (MOI, ratio of PFU over CFU) of approximately 1. Samples were incubated at 30°C until visible cell lysis occurred after approximately 1 or 2h. Subsequently, 1 ml of an exponentially growing culture was added to the tube of infected donor strain culture to increase both the phage titre and the chance for transducing particles to form. This step was performed twice with a one-hour interval. Then, the sample was centrifuged at 3,500 x g for 10 min in an Eppendorf tabletop centrifuge 5810R (Eppendorf, Hamburg, Germany). The supernatant was filter-sterilized using a 0.45 μm filter and stored at 4°C. The bacteriophage titre in the lysate was estimated on the bacterial strain to be used as recipient strain for the transduction experiment. All lysates used in the transduction experiments had a titre ≥ 10^9^ PFU/ml.

An overnight-grown culture of the recipient strain was diluted to 2% in fresh GM17 medium with 10 mM CaCl_2_ and the appropriate antibiotics, and incubated at 30°C until an OD_600nm_ of 0.3 was reached. The culture was subsequently divided into 1- or 10-ml samples according to the MOI values that had to be tested. One 10-ml sample was kept as a control. Cells were harvested by centrifugation at 11,000 x g for 5 min at 4°C in an Eppendorf table top centrifuge 5418 for small volumes or 5810R for bigger volumes, and resuspended in 300 μl of ice-cold 10 mM MgSO_4_. Phage lysate in GM17 was added to each tube to reach the desired MOI after which CaCl_2_ was added to a final concentration of 10 mM. Fresh GM17 medium (100 μl) was added to the control sample. The mixture was briefly vortexed and incubated at RT for 15 min to allow phage adsorption before plating on GM17 agar plates supplemented with the appropriate antibiotics. The plates were incubated for 48 h at 30°C and then examined for the presence of transductant colonies. As a control to ensure that no donor cells were present in the lysate sample, 100 μl of undiluted phage lysate was plated on GM17 agar and on GM17 agar containing the appropriate antibiotics. The frequency of transduction for each MOI used during the infection of the recipient strain was calculated as the number of CFU of transductants per CFU of donor. Each infection, at a specific MOI, was tested in at least two independent experiments.

### Bacteriophage DNA isolation

Phage DNA was isolated starting from 5 ml of phage lysate obtained as previously described. The lysate was mixed with 10% w/v polyethylene glycol MW 800 and 0.5 M NaCl, and incubated for 16 h at 4°C to allow the phage particles to precipitate. The sample was subsequently centrifuged for one h at 11,000 x g at 4°C in an Eppendorf table top centrifuge 5810R (Eppendorf). The phage pellet was resuspended in 400 μl DNAseI buffer (10 mM Tris-HCl, pH 7.6, 2.5 mM MgCl_2_, 0.5 mM CaCl_2_). Residual host DNA and RNA were degraded by incubation at 37°C for at least 30 min with 1μg/ml each of DNAseI and RNAseI (Merk KGaA, Darmstadt, Germany). Ethylendiaminetetraacetic acid (EDTA) was then added at a final concentration of 5 mM and the sample was incubated at 65°C for 15 min to inactivate the enzymes. Phage capsids were degraded by incubating the mixture for 20 min at 56°C with proteinase K (Merk KGaA) at a final concentration of 2 μg/ml. Sodium dodecyl sulfate (SDS) was added to 2.5% v/v and incubation was continued at 65°C for 10 min. Phage DNA was purified by two consecutive phenol/chloroform extractions: the sample (approx. 500 μl) was mixed with an equal volume of a mixture of phenol:chloroform:isoamyl alcohol (25:24:1) in a 2-ml 5-Prime phase lock gel-light tube (Quanta BioScience, Beverly, MA, USA) and centrifuged at 13,000 x g for 8 min in an Eppendorf table top centrifuge 5418. The upper DNA-containing aqueous phase was mixed by inversion with 0.1 volume of 3 M sodium acetate (pH 4.8) and 2.5 volumes of 80% ice-cold ethanol and incubated at -20°C for at least 1 h to allow DNA precipitation. DNA was concentrated by centrifugation at 13,000 x g for 15 min in an Eppendorf table top centrifuge 5418 and washed twice with 1 ml of 80% ice-cold ethanol. The DNA pellet was air dried for at least 1 h before resuspending in 50 μl TE buffer (10 mM Tris-HCl, pH8, 1 mM EDTA). DNA samples were stored at 4°C until use.

### Random transposon library construction

**Fig 1:**
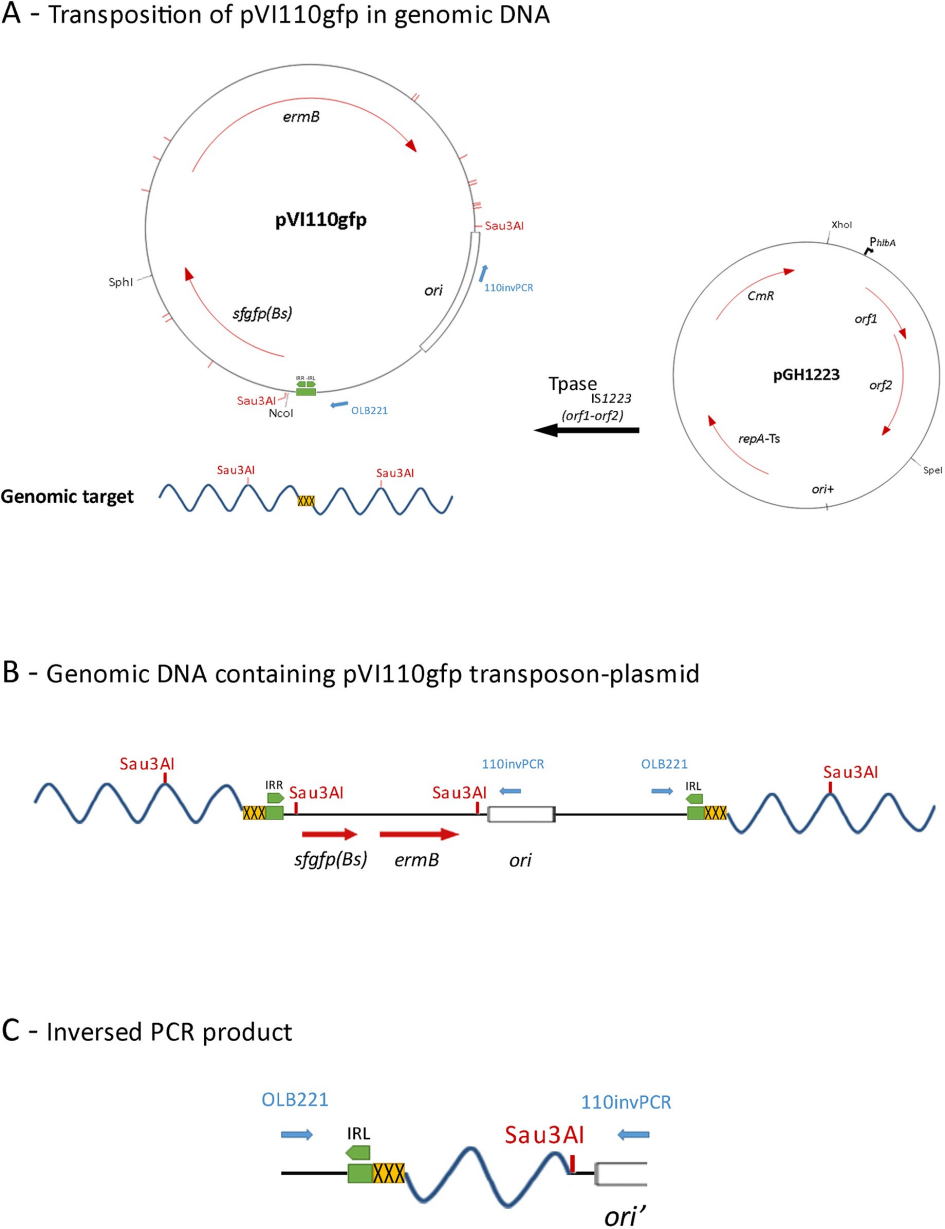
Representation of the transposition mechanism used for the construction of the library in *L*. *lactis* MG1363, and of the analysis of the obtained clones. Panel A: The maps of the two plasmids used for the construction of the library, are shown. The integration of pVI110gfp into the genomic DNA is promoted *in trans* by the action of the IS1223 Tpase, encoded by plasmid pGH1223, on the abutted left (IRL) and right (IRR) inverted repeats of the transposon plasmid (indicated in green in the plasmid map). Blue arrows correspond to the annealing sites of primers OLB221 and 110invPCR on pVI110gfp. The recognition sites of the Sau3AI restriction enzyme are indicated in red. The Genomic DNA is indicated as a blue wavy line and the yellow box indicates the 3 to 4 base pairs of the genomic target site. Panel B: Map of the integrated pVI110gfp. Genomic DNA is represented as a blue wavy line and DNA from the integrated plasmid is shown as a black line. The duplication of the genomic target site (yellow box) that occurs during the transposition process is visible next to the IRL and IRR repeats. Panel C: Representation of inversed PCR product. The inversed PCR fragments, containing a variable length of genomic DNA, are obtained by using primers OLB221 and 110invPCR, and Sau3AI digested and self-ligated pVI110gfp integrated genomic DNA as template.

A random transposon library was constructed in *L*. *lactis* MG1363(+pLP712) based on the previously reported random transposon mutagenesis system P_junc_-TpaseIS*1223* [29]. The suicide transposon plasmid, pVI110gfp (Fig 1A), was constructed by cloning the *sfgfp(Bs)* gene [30] downstream the P_junc_ sequence in pVI110 [29] in order to select for GFP expression of random transposon mutants. The *sfgfp(Bs)* gene was amplified using primers gfpDSM-NcoI-FW and gfpDSM-SphI-RV (Table 3), and pSEUDO::Pusp45-sfgfp(Bs) [30] as template. The obtained PCR fragment was digested using the restriction enzymes NcoI (recognizing C^CATGG) and SphI (recognizing GCATG^C) according to the instructions of the manufacturer (ThermoFisher Scientific, Walthman, MA, USA), and cloned in the corresponding sites of pVI110. The helper plasmid, pGH1223 (Fig 1A), was constructed by inserting the IS*1223* transposase gene of plasmid pVI129 [29] in the temperature sensitive plasmid pVE6007 [31] to force transposition of pVI110GFP and consequent loss of pGH1223, by shifting to the non-permissive temperature (37°C). The transposase gene was amplified using primers GH1223-F-XhoI and GH1223-R-SpeI (Table 3) and pVI129 as a template. The obtained PCR fragment carrying P_hlbA_- IS*1223*ΔIR was subsequently cleaved using restriction enzymes XhoI and SpeI and ligated into the corresponding sites of pVE6007.

**Table 3:** List of primers used in this study.

Primer name	Sequence (5’ → 3’)	Reference
**gfpDSM-NcoI-FW**	GATCCCATGGAGATCTCGAGTACTGATTAACTAATAAGGAGG	This work
**gfpDSM-SphI-RV**	GCGCGCATGCACTAGTGCTCATTATTACTTATAAAGCTCATCC	This work
**GH1223-F-XhoI**	GATCCTCGAGACTGATGCACTTCTCCTACC	This work
**GH1223-R-SpeI**	GATCACTAGTTTTTAAAGATTTGATAATACACG	This work
**OLB221**	AGCTATGCATCCAACGCGTTGGG	[29]
**110invPCR**	AGGATTAGCAGAGCGAGG	This work

pGH1223 was first introduced in *L*. *lactis* MG1363 (+pLP712) via electrotransformation [28] using a Gene Pulser electroporation system (Bio-Rad Laboratories, Richmond, CA, USA). Transformants were selected after O/N incubation at 30°C on LM17 plates supplemented with 0.5 M sucrose and 5 μg/ml Chloramphenicol. The resulting strain was subsequently transformed with pVI110GFP as previously described. The transformed cells were first recovered in GM17 media supplemented with 50 ng/ml Erythromycin for two hours at 30°C to allow expression of the transposase. Transformants were subsequently plated on GM17 plates supplemented with 0.5 M sucrose and 5 μg/ml Erythromycin and incubated at 37°C for 48 hours to induce the loss of plasmid pGH1223 (Fig 1B). A total of approximately 20.000 separate colonies were subsequently resuspended in 16 ml of M17 supplemented with 0.5 M Sucrose and 10% glycerol and stored at -80°C until use.

### Transposon library evaluation

The transposon library evaluation was performed by PCR footprinting and transposon mapping.

First, the total DNA of the transposon library was isolated from 2 ml overnight culture as previously described [32] with the following modifications. Five ml of GM17 medium supplemented with 5 μg/ml Erythromycin was inoculated 1:50 with a glycerol stock (-80°C) of the random promoter transposon library of *L*. *lactis* MG1363 (+pLP712) and incubated overnight at 30°C. The washed cells were resuspended in 0.5 ml of resuspension buffer (10 mM Tris-HCl (pH8.0), 10 mM EDTA (pH 8.0), 50mM NaCl, 20% Sucrose, 5 mg/ml Lysozyme) and incubated for 10 min at 55°C. After the lysis step with SDS, the Proteinase K treated lysate was extracted once with an equal volume of phenol-chloroform-isoamyl alcohol (25:24:1) and once with an equal volume of chloroform-isoamyl alcohol (24:1). After ethanol precipitation, DNA was dissolved and incubated overnight at 37°C in 200 μl 10 mM Tris-HCl (pH8.0) and 1 mM EDTA (pH 8.0) containing 10 mg RNase per ml.

For the inversed PCR, 24 μg of the transposon library total DNA was first incubated overnight at 37°C with the restriction enzyme Sau3AI (recognizing ^GATC) according to the manufacturer instructions (ThermoFisher Scientific) and the digested fragments were subsequently purified using a NucleoSpin Gel and PCR Clean-up kit according to the manufacturer instructions (Macherey-Nagel, Düren, Germany) (Fig 1C). The purified digested fragments were diluted to a final concentration of 1 ng/μl in 48 μl ligation buffer, denatured via incubation at 95°C for 7 minutes and cooled down on ice prior to adding 2U of T4 Ligase (ThermoFisher scientific) to allow for self-ligation. The ligation was performed at 20°C for 60 minutes after which the ligase was inactivated via incubation at 65°C for 10 min.

The inversed PCR was performed on 5ng of the ligation mix using the transposon specific primers OLB221 and 110invPCR, which are located 124 and 147 bp, respectively, from the embedded random genomic DNA fragment (Fig 1B). Taq polymerase was used for the amplification with the following conditions: 95°C for 5 min; 50 cycles of 95°C for 30 sec, 50°C for 30 sec, 72°C for 4 min; and 72°C for 5 min. The inversed PCR reaction was repeated up to 8 times and the PCR products of each reaction were combined and purified using a NucleoSpin Gel and PCR Clean-up kit according to the manufacturer instructions (Macherey-Nagel).

**Fig 2:**
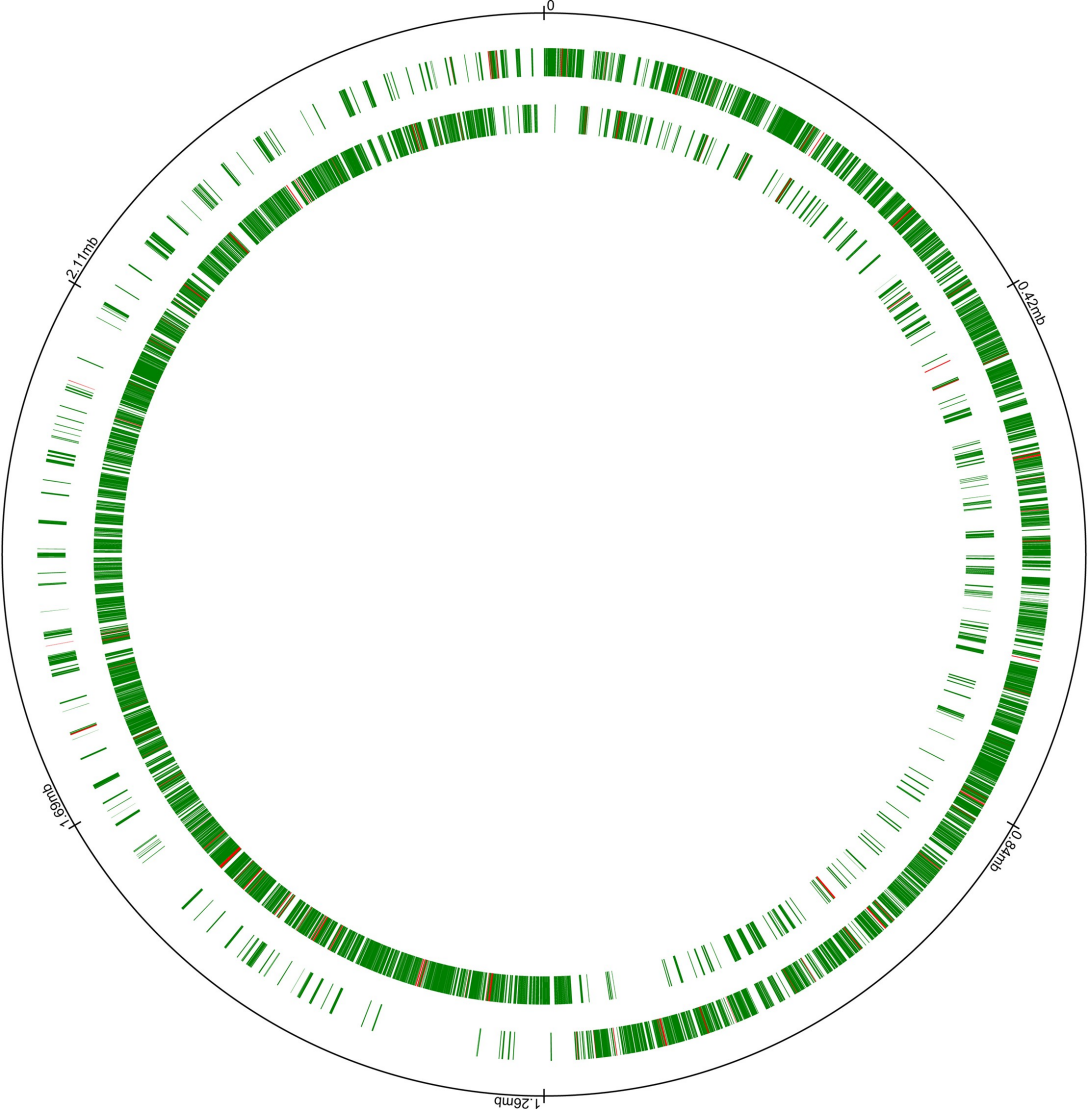
Map of transposon integration in *L*. *lactis* MG1363 genome. The rings represent, from outside to inside: genomes coordinates; ORFs on the plus strand interrupted by the insertion of pVI110gfp (green) and not interrupted by the insertion of pVI110gfp (red); ORFs on the minus strand interrupted by the insertion of pVI110gfp (green) or not interrupted by the insertion of pVI110gfp (red). The map was generated using *Civi* [37].

Approximately 16 μg of inversed PCR product was sequenced to map the transposon library. The sequence data was processed to eliminate the transposon specific sequences and insertion sites of intergenic regions to analyze and map the *L*. *lactis* MG1363 genes interrupted by the insertion of pVI110GFP (Fig 2). Since the library was used only for chromosomal DNA transfer analysis, the transposon insertion sites on pLP712 were not analyzed.

### Bioinformatics analyses

The nucleotide sequences of bacteriophage genomes were determined using the Illumina MiSeq platform with 2 x 150-bp paired-end sequencing (Illumina, San Diego, CA, USA). Nucleotide sequences were trimmed, analysed and assembled using the A5-Myseq pipeline [33] and the assembled contigs were annotated using the RASTtk server [34]. The end termini of the phage genomes were predicted using the PhageTerm tool [35]. Local sequence alignments were generated using the Smith Waterman algorithm [36]. The *L*. *lactis* MG1363 genome map was visualized using *Civi* [37].

### Genbank accession numbers

The annotated genome sequences of all the bacteriophages used in this work are deposited in GenBank (Table 2).

## Results

### Three strictly lytic lactococcal bacteriophages were selected to investigate generalized transduction

A collection of bacteriophages isolated over the past three decades from dairy factories in different parts of the world was used to select a group of phages to employ for our transduction experiments (Table 2). In an attempt to develop a general tool for phage-mediated gene transfer that can be used in industrial settings, we decided to focus on generalized transduction only. For this reason, we discarded phages belonging to the Bk5-t species as these phages are known to be temperate [38]. The second important parameter that we used for bacteriophage selection was their ability to infect the plasmid-free laboratory strain *Lactococcus lactis* MG1363. Using this strain would allow employing the well-characterized plasmids and genetic tools that have been developed for *L*. *lactis* in the past decades. Of the six phages infecting strain MG1363 and belonging to the c2 species (and therefore strictly lytic), CHPC966, 5171F and 5105F were selected for further analyses (Table 2).

### Chromosomal DNA transfer via lytic bacteriophages could not be detected

As a first step, we investigated the ability of the selected bacteriophages to transfer chromosomal DNA via generalized transduction. A transposon library constructed in *L*. *lactis* MG1363 (pLP712) was employed in this case. Each clone of the library harbours a cassette containing a copy of the Ery^r^ gene and a promoterless GFP gene in its chromosome. The transposon mutants were selected as erythromycin-resistant GFP^+^ colonies and, thus, carry the cassette downstream of an active promoter (unpublished internal laboratory results). Utilizing the Ery^r^ and/or GFP^+^ phenotypes as selectable markers should allow identifying generalized transduction events. Cultures of the total library were infected with each of the three phages under examination, at an MOI ≥ 1 in order to obtain phage lysates that would contain, among the wild-type phages, transducing particles carrying DNA of bacterial origin. The resulting lysates were used to infect the recipient strain *L*. *lactis* MG1614, a plasmid -free Str^r^-Rif^r^ derivative of *L*. *lactis* MG1363 [39], at a range of MOI values between 0 and 1. Transductant colonies were, however, never obtained with any of the bacteriophages under any of the conditions analysed.

Given these results and the intrinsic technical limitations of using a library of donor strains (e.g. non homogeneous propagation of clones, insertion of the transposon into essential genes) we cannot speculate on the feasibility of using bacteriophages CHPC966, 5171F and 5105F, or any other strictly lytic lactococcal phage, to mobilize chromosomal DNA between lactococcal strains.

### Plasmid DNA transfer depends on the phage utilized and on the multiplicity of infection

We next decided to test the ability of the three phages of transducing plasmid DNA. To this end, *L*. *lactis* MG1363 (pNZ8048) was used the donor strain. Plasmid pNZ8048 is a 3.4-Kb high-copy number lactococcal expression vector [40] harbouring a chloramphenicol resistance (Cm^r^) gene that was used as selectable marker to identify transductant cells. *L*. *lactis* strain MG1614 was chosen as the recipient strain and a range of MOI values between 0 and approx. 1 was employed. Plasmid DNA from putative transduced colonies was extracted and subjected to restriction enzyme digestion to verify plasmid identity. All colonies obtained and analysed in this way carried the pNZ8048 plasmid with no deletions or DNA insertions, proving that they were genuine transductants (S1 Fig).

**Fig 3:**
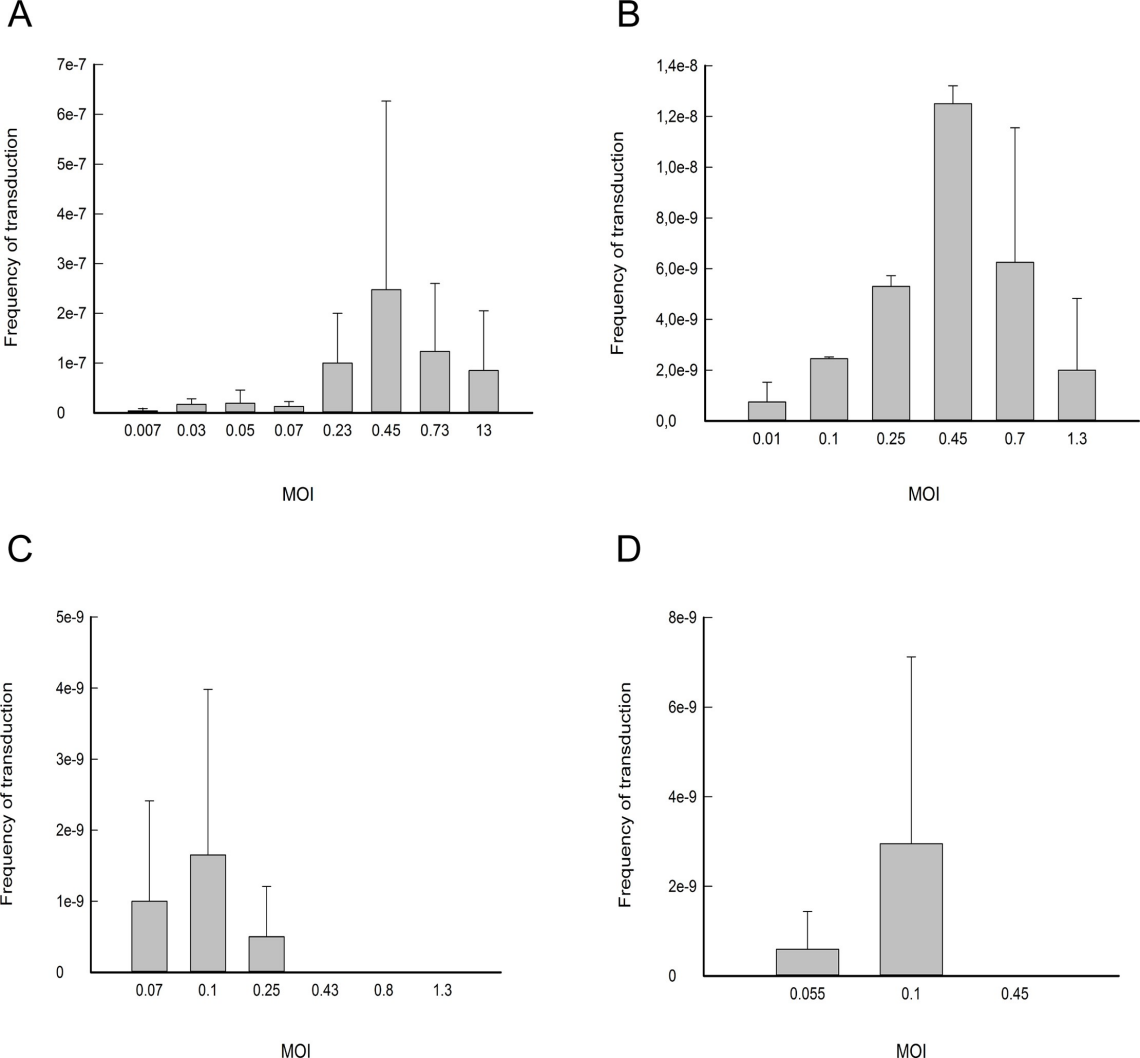
Frequency of plasmid transduction by bacteriophages analysed in this study. Panels A, B and C show the results of transduction of pNZ8048 using bacteriophage CHPC966, 5171F, or 5105F respectively. Each infection, at a specific MOI, was tested in at least two independent experiments, the results reported show the average result for each MOI, and the corresponding standard deviation. Panel D: transduction of pGKV552 via phage CHPC966. The frequency of transduction (Y-axis) for each MOI used during the infection of the recipient strain (X-axis) is calculated as the number of colony forming Units (CFU) of transductants per CFU of donor.

The results of pNZ8048 transduction by the bacteriophages CHPC966, 5171F and 5105F are shown in Fig 3. The optimum transduction frequency occurs at an MOI well below 1 for all three phages. The data also show that, at comparable MOI values, plasmid transduction frequencies differed among the phages. For example, the highest frequency of transduction was reached when the recipient strain was infected at an MOI of 0.4 with either bacteriophage CHPC966 or 5171F (Fig 3, panel A and B, respectively) but plasmid transfer at this MOI was 10-fold higher using phage CHPC966. When bacteriophage 5105F was employed, on the other hand, the highest transduction frequency was registered at an MOI of 0.1, while no transductants were obtained when the recipient was infected at an MOI of 0.4 (Fig 3, panel C). Moreover, the highest transduction frequency reached with this phage was approximately 10^−9^, which is almost 100 and 10-fold lower than the highest frequency obtained with phages CHPC966 and 5171F, respectively.

Taken together these results demonstrate that different lytic lactococcal bacteriophages are able to transfer plasmid pNZ8048 at widely different efficacies. Not only does the number of transductants obtained depend on the MOI at which the recipient strain is infected, but different bacteriophages, tested under the same experimental conditions, transfer the same plasmid at different frequencies. Finally, the highest transduction frequency can vary dramatically depending on the bacteriophage used.

### Plasmid transduction frequencies vary when different plasmids are used

In order to examine at which rates a given bacteriophage can transduce different plasmids, we investigated the ability of phage CHPC966 to transfer plasmid pGVK552 under the conditions used for pNZ8048 transduction. Plasmid pGKV552 is an 11,8-Kb, low-copy number plasmid, carrying an erythromycin resistance (Em^r^) gene, which was used as marker to select transductants, and the industrially important proteinase gene *prtP* and maturase gene *prtM* [41].

**Fig 4:**
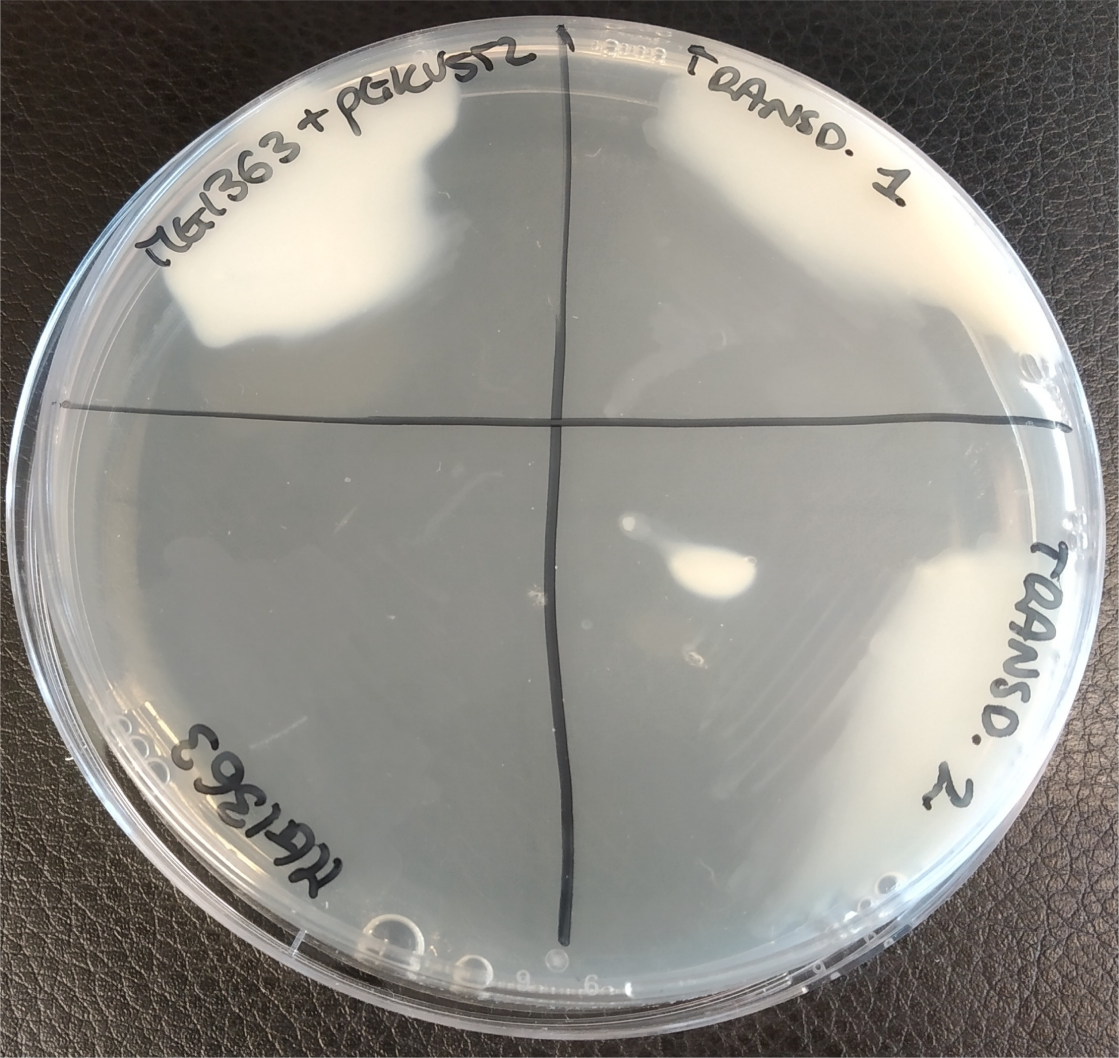
Proteinase activity plate assay. Growth on skim milk agar plates of *L*. *lactis* strains MG1363, MG1363 (pGKV552) and two representative transductants obtained from pGKV552 transduction mediated by phage CHPC966. The sodium citrate in the plate leads to a transparent medium. The ability of a strain to hydrolyse casein and to, consequently, grow and produce acid leads to opaque bacterial growth.

Restriction enzyme analyses on plasmid DNA from putative transductants revealed that all 10, except one, carried intact pGKV552 plasmid (S1 Fig). Furthermore, the PrtP proteinase activity of the isolated transductants was examined by growing the strains on skim milk plates supplemented with glucose as carbon source. Nine of the 10 transductants could grow on these plates while the plasmid fee parental strain could not (Fig 4). Growth of *L*. *lactis* on these plates depends on casein breakdown to liberate essential amino acids. These results confirm that the *prtPM* genes are intact in 9 out of 10 transductants, leading to synthesis of a functional proteinase and showing that pGKV552 could be successfully transferred during phage transduction. We hypothesize the only transductant that could not grow on skim milk plates, to be a spontaneous erythromycin resistant strain that does not carry, in fact, the pGKV552 plasmid.

The results of the transduction experiment are shown in Fig 3D. The highest frequency of transduction was ca. 3x10^-9^ and was obtained at a MOI of 0.1, while no transductants were obtained at higher MOI values. Transfer of pGVK552 by phage CHPC966 was almost 100-fold lower than that of plasmid pNZ8048. These results show that, under similar conditions, phage CHPC966 can transduce different plasmids at different frequencies. Although the frequencies of transduction registered for plasmid pGKV552 are lower than those for pNZ8048, it is worth mentioning that pGKV552 is more than 3 times bigger than pNZ8048 and that it represents half the size of phage CHPC966 genome. It is tempting to speculate that phage CHPC966 could be used to transfer even bigger plasmids with even more industrially relevant genes.

### Plasmid sequence analysis and their involvement in transduction frequency

Using the PhageTerm tool we examined the genome sequences of the three bacteriophages used in this study. This analysis revealed that they all possess cohesive (*cos*) ends with the following predicted sequences: 5’-ATCAAGCCTAACT-3’ (for phage CHPC966) and 5’- CAAGCCNNNNT- 3’ (for phages 5171F and 5105F). It has previously been demonstrated that cloning the *cos* sequence of a certain phage genome into a plasmid, facilitates the transduction of the modified plasmid by phage [42, 43]. For this reason we decided to search, in pNZ8048 and pGKV552, for the presence of sequences identical to the bacteriophages *cos* sequences, that could have played a role in the positive outcome of the tested transduction events. For this purpose we performed an *in silico* local alignment between the plasmids and the *cos* nucleotide sequences, using the Smith Waterman algorithm. The results of this analysis are shown in S1 Table. The low accuracy of phages 5171F and 5105F *cos* sequence prediction, and the lack of identical hits between the two plasmids and phage CHPC966 *cos* sequence, make it difficult to infer whether the plasmids nucleotide sequence influenced the transduction frequencies reported in this study.

## Discussion

Bacteriophage transduction is a naturally occurring event that contributes to HGT among bacteria [15]. We investigated this process in *L*. *lactis* with the aim of assessing whether it could be exploited as a reliable methodology for *L*. *lactis* genome editing. Three different strictly lytic lactococcal bacteriophages were examined with respect to their potential to transfer plasmid and/or chromosomal DNA between *L*. *lactis* strains. The plasmids employed in this study contain antibiotic resistance markers, as they were only used to provide proof of principle. While chromosomal DNA transfer could not be observed under any of the tested conditions, plasmid DNA transduction was established with all three bacteriophages. As we show, the frequency of plasmid transduction depends on three different factors: (i) the bacteriophage employed, (ii) the DNA fragments transferred, and (iii) the multiplicity of infection (MOI) used on the recipient strain.

Our results demonstrate that a specific plasmid is transferred at different frequencies by different phages even when all the experimental parameters are comparable. The size of the plasmids examined here is smaller than that of the genomes of the phages employed and, thus, more than one copy of both plasmids can be accommodated inside the capsid of all three bacteriophages. For this reason, we speculate that plasmid size is unlikely to play a major role in the differences in transduction frequencies. Our *in silico* analysis of the *cos* sequences of the three bacteriophages and of the two plasmids, revealed that no *cos-*identical phage sequences are present inside the transduced plasmids. A few similar sequences were, however, identified (S1 Table). Since the binding accuracy of the phage terminases to the *cos* sequences is not known for the phages used here, we cannot rule out that the identified *cos-*similar sequences in the plasmids did influence the frequency of transduction. Possibly another factor played a more important role in the transduction outcome namely the 10-fold difference in the copy numbers of the two plasmids, approximately 50 and 5 for pNZ8048 and pGKV552 respectively [40, 41]. It seems logical that with a higher number of plasmid molecules inside the cell, the possibility for some of these molecules to be encapsulated into nascent phage particles is higher, increasing the rate of transduction. Both plasmids were most probably actively replicating during infection of the exponentially growing plasmid-containing donor strain by phage CHPC966. As both pNZ8048 and pGKV552 replicate using the rolling circle mechanism [40, 41], we hypothesize that the concatemeric plasmid replication intermediates resemble the bacteriophage genome replication products close enough to allow the phage terminases to recognize and package them into nascent phage heads.

It also appears that the MOI at which the recipient strain is infected is of crucial importance for successful transduction to take place. The MOI during bacteriophage infection is defined as the number of infecting particles divided by the number of bacterial cells, thus expressing the average number of phages present per bacterial cell in an infection mixture. The higher the MOI, the higher the chance that a single cell is infected by more than one phage (S2 Fig). As the aim of this investigation was to exploit bacteriophage transduction for bacterial genome editing, it was imperative to avoid multiple infection of a single recipient cell during infection with the transducing phage lysate. If a cell is infected by a transducing phage particle and at the same time by an intact phage, the latter would lead to phage progeny being produced and, ultimately, result in the burst of the cell and the consequent loss of the transductant. Therefore, we primarily tested MOI values ≤ 1 and observed that, as expected, the frequency of transduction reached a (phage-specific) maximum at a certain MOI value below one.

To the best of our knowledge, chromosomal DNA transduction mediated by lactococcal lysogenic phages has only been reported once [44]. During specialized transduction, chromosomal DNA coupled to part of the prophage genome is mobilized, leading to the integration of viral genes into the chromosome of the recipient strain. It has been proven in other organisms that excision of the truncated prophage, with consequent rearrangements of the neighbouring host chromosomal genes, or even phage-induced cell lysis, could still occur in recipient strains during subsequent cell division cycles [45]. In an industrial scenario, these events could modify the metabolic abilities of the starter strain or lead to starter lysis and failure of the fermentation process and it is, therefore, to be avoided when phage transduction is employed in an industrial setting.

Thus, we examined strictly lytic bacteriophages for their ability to transfer chromosomal DNA but could not, under any of the conditions tested, isolate colonies of transduced cells.

It is unknown whether or how lytic lactococcal bacteriophages hydrolyse the chromosome of the host during their replication cycle. This process is most probably required for chromosomal DNA fragments to be erroneously packaged into phage particles. Only *L*. *lactis* bacteriophage c6A has been proven to encode a DNA endonuclease, which it seems to use to degrade host chromosomal DNA upon infection [46]. The genome sequence of this phage, and so also of this specific endonuclease, is not available and it is not possible to evaluate whether other lactococcal phages specify the same enzyme activity. Examining the nucleotide sequences of lactococcal bacteriophages in the public database as well as those of the phages used in this study, revealed that none of their predicted ORFs encode a known DNA endonuclease. If the bacteriophages tested here indeed do not degrade host chromosomal DNA, this would dramatically lower the chances of chromosomal DNA transduction.

Other aspects of the experimental setup could, of course, also have caused this negative outcome. Firstly, given the protocol used to generate the library, some clones could be underrepresented in the total pool of donor cells decreasing the chance of transduction of the specific part of the chromosome represented in those clones. Secondly, insertion of the transposon inside an essential gene or regulatory region during library construction would lead to the loss of a certain group of clones, lowering the chances of selecting for the transduction of those specific portions of the genome. Lastly, for a chromosomal DNA fragment to be maintained by the recipient strain, double crossover recombination must occur between the host-derived chromosomal DNA fragment from the transducing particle and the resident chromosome. Infection and double crossover recombination are both low probability events lowering the chance of obtaining a transductant for a chromosomal DNA marker.

## Conclusion

In this study, specific cases of generalized transduction mediated by lytic lactococcal bacteriophages were analysed with the aim of assessing whether this horizontal gene transfer mechanism can be used for genome editing of industrial lactococcal strains. Although we were not able to detect generalized transduction of chromosomal DNA fragments, we cannot conclude that this event never takes place during lytic lactococcal phage infections. More detailed and specific studies would be needed in order to verify the feasibility of using phage transduction for this purpose. We can, however, conclude that plasmid transduction is possible under specific circumstances. The selected phage should, of course, be able to infect both the donor and the recipient strains and encode a terminase that can bind the plasmid DNA and package it into nascent phage heads. Consequently, knowing the sequence of the plasmid and the phage genome, or at least the termini of it, would increase the rate of success of the experiment. Isolation of the proper bacteriophage would probably represent the limiting step as that could prove to be a rather long and difficult procedure. Nevertheless, when a plasmid carrying industrially relevant traits is not transferrable via conjugation or mobilization, phage transduction could represent a valid alternative.

## Supporting information

S1 Fig Restriction enzyme profiles of plasmids pNZ8048 and pGKV552 isolated from different transductants obtained in this study. Plasmid pNZ8048 was digested with enzymes SalI and HindIII; expected DNA fragment sizes: 2kb and 1.3 kb. Plasmid pGKV552 was digested with enzymes XbaI and BglII; expected DNA fragment sizes: 7.7 kb and 4.2 kb. First (unmarked) lane of each gel: 1-kb DNA size marker (Thermo Scientific). Lanes 1 and 10, restriction profile of pNZ8048 isolated from *L*. *lactis* MG1363 (pNZ8048). Lanes 2 to 14: restrictions of the plasmids isolated from four representative transductants obtained using bacteriophage CHPC966 (lanes 2–4), bacteriophage 5105F (lanes 6–9) or bacteriophage 5171F (lanes 11–14). Lanes 15: restriction profile of plasmid pGKV552 isolated from *L*. *lactis* MG1363 (pGKV552). Lanes 16 to 19: restriction profile of the plasmid isolated from four representative transductants obtained using bacteriophage CHPC966.
(TIF)

S2 FigPoisson distribution describing the proportion of cells in a population infected with a certain number of phages, at MOIs ranging from 0.1 to 10.The proportion of cells that will be attacked by a specific number of phage particles can be calculated using a Poisson distribution: $P(n)=\frac{m^{n}∙e^{-m}}{n!}$ Where: P(n) is the probability that a cell will be infected by n phage(s), m is the multiplicity of infection, and n is the number of phages infecting a cell.(TIFF)

S1 Table Best hits resulting from the alignment of the bacteriophages predicted *cos* sequence with pNZ8048 and pGKV552 sequences. (DOCX)
