# Supplementary figures and images for: Employing lytic phage-mediated horizontal gene transfer in Lactococcus lactis
Source: PLoS One. 2020 Sep 14;15(9):e0238988. doi: 10.1371/journal.pone.0238988 (PMC7489543; doi:10.1371/journal.pone.0238988)

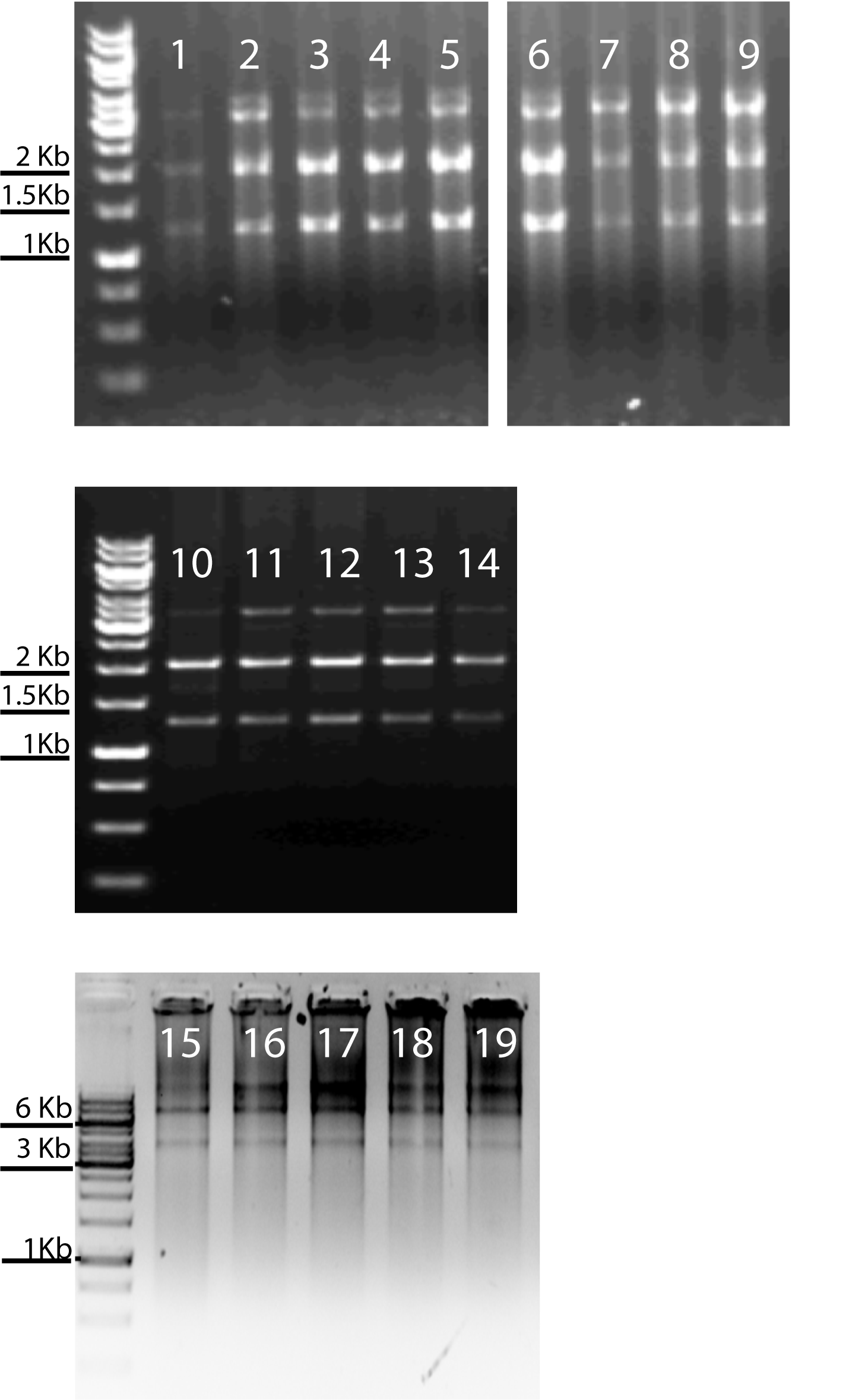

Supplement: S1 Fig — Plasmid pNZ8048 was digested with enzymes SalI and HindIII; expected DNA fragment sizes: 2kb and 1.3 kb. Plasmid pGKV552 was digested with enzymes XbaI and BglII; expected DNA fragment sizes: 7.7 kb and 4.2 kb. First (unmarked) lane of each gel: 1-kb DNA size marker (Thermo Scientific). Lanes 1 and 10, restriction profile of pNZ8048 isolated from L. lactis MG1363 (pNZ8048). Lanes 2 to 14: restrictions of the plasmids isolated from four representative transductants obtained using bacteriophage CHPC966 (lanes 2–4), bacteriophage 5105F (lanes 6–9) or bacteriophage 5171F (lanes 11–14). Lanes 15: restriction profile of plasmid pGKV552 isolated from L. lactis MG1363 (pGKV552). Lanes 16 to 19: restriction profile of the plasmid isolated from four representative transductants obtained using bacteriophage CHPC966. (TIF) [file pone.0238988.s001.tif]
