# Supplementary material for: Employing lytic phage-mediated horizontal gene transfer in Lactococcus lactis
Source: PLoS One. 2020 Sep 14;15(9):e0238988. doi: 10.1371/journal.pone.0238988 (PMC7489543; doi:10.1371/journal.pone.0238988)
Supplement: S1 Table — (DOCX) [file pone.0238988.s003.docx]

**S1 Table.** **Best hits resulting from the alignment of the bacteriophages predicted *cos* sequence with pNZ8048 and pGKV552 sequences.**

| Plasmid | Bacteriophage under examination | Alignment with predicted phage *cos* sequence (5’🡪 3’)^a^ | Strand | Position of the sequence in the plasmid^b^ |
| --- | --- | --- | --- | --- |
| pNZ8048 | CHPC966 | ATTAGGCCTATCT  **ATCAAGCCTAACT** ** * ***** ** | - | *cm^r^* |
| pNZ8048 | 5171F - 5105F | CAAGCCTTGGT  **CAAGCCNNNNT**  ****** * | - | Non coding region |
| pGKV552 | CHPC966 | AACAACCCTAACT **ATCAAGCCTAACT** * *** ******* | - | *prtP* |
| pGKV552 | CHPC966 | ATAAAGTCTAACA **ATCAAGCCTAACT**  ** *** ***** | + | Non coding region |

^a^ The predicted *cos* sequence of bacteriophage in exam is indicated in bold underneath the aligned sequence in the analysed plasmid. * (asterisk) indicates position of fully conserved residue, no entry indicates non-conserved residues.
^b^ *cm^r^*= chloramphenicol resistance gene.
*prtP* = proteinase gene.
